# Performance of porous carbons based on waste-printed paper on the CO_2_ capture by temperature swing adsorption

**DOI:** 10.1007/s11356-026-37526-0

**Published:** 2026-03-09

**Authors:** Yanier Sánchez-Hechavarría, Jorge Arce-Castro, João Pedro Santos Alves, Tamille Alves Souza, Mauricio Brandão dos Santos, Fernanda Teixeira Cruz, Raildo Alves Fiuza Junior, Karen Valverde Pontes, Artur José Santos Mascarenhas

**Affiliations:** 1 Institute of Chemistry, Department of General and Inorganic Chemistry, Laboratory of Catalysis and Materials (LABCAT), Federal University of Bahia, Trav. Barão Do Jeremoabo, 147, Campus de Ondina/Federação, Salvador , 40170-280 Bahia Brazil; 2 Post-Graduation Program in Energy and Environment (PGENAM), Polytechnic School of Engineering, Federal University of Bahia, R. Prof Aristides Novis, 2, Federação, Salvador, 40120-910 Bahia Brazil; 3 Polytechnic School of Engineering, Department of Chemical Engineering, Laboratory of Sustainaible Processes and Renewable Energy (LAPSER), Federal University of Bahia, R. Aristides Novis, 2, Federação, Salvador, Bahia 40210-630 Brazil; 4 Post-Graduation Program in Chemistry (PGQuim), Institute of Chemistry, Federal University of Bahia, Travessa Barão de Jeremoabo, 147, Campus Ondina, Salvador, Bahia 40170-280 Brazil; 5 National Institute of Science and Technology in Energy and Environment (INCT-E&A), Interdisciplinary Center of Energy and Environment (CIENAM), Federal University of Bahia. Rua Barão de Jeremoabo, S/N, Campus de Ondina, Salvador, Bahia 40170-115 Brazil

**Keywords:** Waste, Printed paper, Activated carbon, CO_2_adsorption, Temperature swing adsorption (TSA), Adsorption kinetics

## Abstract

The development of low-cost and sustainable adsorbents is crucial for reducing CO_2_ emissions associated with climate change. In this context, waste-derived porous carbons have emerged as promising materials for adsorption applications. This work evaluates the CO_2_ adsorption performance of porous carbons derived from printed paper waste using temperature swing adsorption (TSA). The activated carbons were produced by pretreating printed paper waste, followed by impregnation with KOH, K_2_CO_3_, or KHC_8_H_4_O_4_ and chemical activation via pyrolysis at 800 °C. Adsorption tests were conducted using TSA, assessing adsorption capacity as a function of temperature. The results showed that the maximum CO_2_ adsorption capacity at 30 °C for AC-KOH, AC-KCO, and AC-KHP was 7.64%, 6.28%, and 6.80%, respectively. In adsorption tests at 30 °C at 1 bar, AC-KOH (83.33% micropore volume) demonstrated the best performance with a maximum CO_2_ adsorption efficiency of 7.64% (2.29 mmol g^−1^). The average fluctuation after 10 adsorption–desorption cycles was less than 1%, indicating good regeneration and stability of AC-KOH. These results suggest that printed paper is a promising precursor for producing adsorbents for CO_2_ adsorption.

## Introduction

In urban solid waste, paper of different types constitutes one of the most significant fractions. Used paper is an essential component of this waste, with an annual production exceeding 300 million tons worldwide (Putro et al. 2019). The complexity of recycled paper lies in its composition, which involves cellulose fibers (containing polar functional groups such as alcohol and ether) and inorganic fillers, such as kaolin, clay, and calcium carbonate (Mabalane et al. 2024).

Traditionally recycled for the production of new paper, paper has gained new applications in the last decade (Zhang and Zhu 2021), serving as raw material for cellulose derivatives and bioethanol. The production of recycled fibers from paper is mostly carried out through the conventional process of removing ink by chemical flotation, a well-established and extensively documented method. In parallel, alternative approaches such as the use of enzymes, ultrasound, and adsorbents show potential, although their large-scale implementation in paper recycling units is still incipient. The effectiveness of these different methods is directly related to the complex interaction between the characteristics of the paper and the printing inks used (Vukoje and Rožić 2018; Inkoua et al. 2022).

Aiming at the mitigation of CO_2_ emissions, the application of activated carbons from plant biomass in capture and adsorption processes is an area of growing interest. In this scenario, the valorization of paper and cardboard waste for the production of low-cost and highly selective activated carbons for CO_2_ presents itself as a promising solution for sustainable waste management and in line with the principles of the circular economy.

Chemical activation is fundamental to enhance the properties and adsorption capacities of typically carbonaceous materials. In this process, the adsorbent reacts with various agents, such as oxidants, acids, bases, inorganic salts, and organic solvents. However, given the significant environmental pollution generated, the use of environmentally friendly and safe solvents for living organisms becomes imperative (Nguyen et al. 2021; Wei et al. 2024). Chemical activation consists of a thermochemical method that transforms carbonaceous material into activated carbon, condensable volatiles (called bio-oil when the raw material is biomass), and fuel gases (Inkoua et al. 2022).

Recent reviews and studies have shown that chemical activation using agents such as KOH, K_2_CO_3_, and ZnCl_2_ can effectively tailor the microporosity and surface chemistry of activated carbons, resulting in high CO_2_ adsorption performance at low pressures. These methods can produce materials with well-developed narrow micropores and high surface areas suitable for CO_2_ capture applications and are compatible with scalable production processes, while physical activation with CO_2_ may require harsher conditions and often yields broader pore size distributions that are less optimal at near-ambient conditions (Cordeiro et al. 2024; Zentou et al. 2025; Dayi et al. 2025; Kundu et al. 2024).

In the chemical activation of porous carbons from paper and cardboard waste, agents such as H_3_PO_4_ (Adinaveen et al. 2016; Nowrouzi et al. 2018; Xu et al. 2014), K_2_CO_3_ (Ma et al. 2018; Kim et al. 2020; Liu and Huang 2018), ZnCl_2_ (Chang et al. 2019), and K_2_C_2_O_4_ (Sevilla et al. 2018; Rehman et al. 2022) are used, with KOH being the most prevalent (Tseng and Tseng 2005; Chang et al. 2019; Liu et al. 2018; Durairaj et al. 2019; Nahar et al. 2023; Jinyang et al. 2024). KOH induces internal corrosion in the carbon, forming micropores and increasing the surface area as a function of the KOH/carbon ratio. The formation of micro- and mesopores during activation results from the simultaneity of surface pyrolysis and internal corrosion, processes driven by cracking and gasification reactions that remove significant amounts of oxygen, hydrogen, and carbon (Tseng and Tseng 2005).

Despite the prevalence of KOH, a study by Tay et al. (2009) revealed that K_2_CO_3_ provided higher yields in the production of activated carbon from the pyrolysis of soybean oil cake. Furthermore, under the same experimental conditions, the activated carbon derived from K_2_CO_3_ exhibited a higher specific surface area than that produced with KOH (Heidarinejad et al. 2020). Other benefits pointed out by Tay et al. (2009) are the lower ash and sulfur content in the activated carbon obtained with K_2_CO_3_ compared to that produced with KOH. Foo and Hameed (2012) also found that the adsorptive capacity of activated carbon from orange peel increased with the increase in the proportion of K_2_CO_3_ used. Additionally, Adinata et al. (2007) demonstrated that the use of K_2_CO_3_ prevented the formation of tar and other liquid byproducts, such as acetic acid and methanol, during the activation process.

In temperature swing adsorption processes for CO_2_, the search for sustainable carbonaceous materials has directed attention towards biomass waste. Surprisingly, the potential of porous adsorbent carbons produced from paper has not yet been widely investigated in these applications. The studies by Vazhayal et al. (2020) and Jinyang et al. (2024) explored the use of paper and cardboard waste as CO_2_ adsorbents.

Vazhayal et al. (2020) developed a microporous carbon from silk paper waste and polyvinyl alcohol, using a combination of freeze-drying and carbonization. The resulting aerogels exhibited a highly interconnected three-dimensional porous structure, low density (0.057 g cm^−3^), high porosity (~ 97%), and significant specific surface area (1384 m^2^ g^−1^), with a predominance of ultramicropores. These characteristics conferred great potential for CO_2_ adsorption (5.14 mmol g^−1^ at 0 °C) to the material, demonstrating good selectivity and regeneration capacity, without the need for chemical activation, functionalization, or heteroatom doping.

Jinyang et al. (2024) produced hierarchical porous carbon aerogels from various paper wastes, aiming for their application in CO_2_ adsorption. Activation with KOH was performed in a single step, optimizing energy consumption compared to two-step methods. The aerogels derived from printing paper, napkins, and corrugated paper, pyrolyzed at 700, 800, and 900 °C, exhibited specific surface areas between 958 and 1370 m^2^ g^−1^, with a micropore contribution to the surface area ranging from 89.8% to 94.3%. The maximum CO_2_ adsorption capacity at 25 °C and 1 bar for the activated carbons obtained at 800 °C from different paper types ranged between 143 mg g^−1^ (3.25 mmol g^−1^) and 151 mg g^−1^ (3.43 mmol g^−1^), with a CO_2_/N_2_ selectivity of 8 to 11.

In other studies, in which commercial activated carbon was impregnated with NaOH, the material exhibited an average specific surface area of approximately 1022 m^2^ g^−1^, a cumulative pore volume of 0.4123 cm^3^ g^−1^, and an average pore width of 1.543 nm (Nwaisr et al. 2024). The adsorption capacity of this activated carbon for a gas mixture containing 10 mol% CO_2_ in N_2_O reached 290.62 mg g^−1^, which is approximately twice that of the commercial activated carbon prior to impregnation, corresponding to an increase of about 150% relative to the raw material, thereby demonstrating the effectiveness of the impregnation process in enhancing adsorptive performance.

In this study, activated carbons were synthesized from printed paper waste using chemical activation with KOH (potassium hydroxide), K_2_CO_3_ (potassium carbonate), or KHC_8_H_4_O_4_ (potassium hydrogen phthalate, KHP) as agents. The resulting materials were characterized, and the CO_2_ adsorption capacity by temperature swing adsorption and the selective capture of CO_2_ in a N_2_/CO_2_ flow were evaluated. Additionally, the effect of activating agents on the structure of the activated carbons was investigated, contributing to the knowledge about the synthesis and application of sustainable carbonaceous adsorbents in CO_2_ capture.

## Materials and methods

### Preparation of activated carbons

The raw material used in this study consisted of postconsumer office paper (A4, 80 g m^−2^). This specific waste stream was selected for its high cellulose content (approx. 60%–70%) and relatively low lignin content compared to newsprint or cardboard. The printed paper waste was previously cut and ground using a knife mill and subsequently sieved through an 80-mesh sieve.

The production of activated carbons started with a three-stage pretreatment of waste-printed paper (W-PP). In the first stage, the shredded cellulosic material was submerged in a hydroalcoholic solution (50% v/v ethanol) at 60 °C, under magnetic stirring for 120 min, aiming to obtain a homogeneous suspension. This suspension was then subjected to centrifugation to separate the solid and liquid phases. The second stage consisted of immersing the resulting solid material in deionized water at 80 °C, with magnetic stirring for 120 min. The third stage involved washing the cellulosic pulp with deionized water at room temperature, followed by vacuum filtration to remove excess liquid. After the pretreatment, the wet pulp was chemically impregnated using an aqueous solution of the activating agents KOH, K_2_CO_3_, or KHC_8_H_4_O_4_ in a mass ratio of 1:1 (activator/precursor). This mass ratio was defined based on the suggestion of Gurten et al. (2012) for activated carbons prepared using waste tea leaves and K_2_CO_3_. For each activation process, 30 g of paper pulp and 30 g of the activating agent were weighed, corresponding to a 1:1 mass ratio, and dispersed with 38.6 g of deionized water. The subsequent evaporation of water and drying of the impregnated material in an oven at 100 °C for 12 h completed the activation step.

The carbonization step of the three impregnated materials was conducted in a tubular furnace at 800 °C under a nitrogen atmosphere with a flow rate of 150 mL min^−1^ and a controlled heating rate of 10 °C min^−1^. The selection of the carbonization temperature for the paper pulp obtained from treated paper waste followed the recommendations reported by Liu et al. (2018), Ma et al. (2018), Sundriyal et al. (2021), and Son et al. (2024). These studies employed a carbonization temperature of 800 °C and reported excellent results in terms of structural characteristics and adsorption properties of the resulting activated carbons. In addition, the thermogravimetric analysis of the paper waste was considered, showing that at 800 °C the thermogravimetric profile reaches a plateau, which indicates the completion of the carbonization process and the effective removal of unstable volatile matter.

The resulting activated carbons were subjected to a washing process with a 0.1 mol L^−1^ aqueous HCl solution at 80 °C, aiming at neutralization and removal of residual inorganic species, followed by successive washes with deionized water at room temperature until a neutral effluent (pH = 7.0) was obtained. The final drying of the materials was carried out in an oven at 110 °C for a period of 12 h. The resulting activated carbons were ground and sieved to a particle size range of 100–200 mesh (75–150 μm).

The activated carbons obtained through the described methodology were identified as AC-KOH (KOH), AC-KCO (K_2_CO_3_), and AC-KHP (KHC_8_H_4_O_4_). Additionally, a reference carbon (RC-PP) was prepared by carbonizing waste printed paper not subjected to chemical activation, using the same carbonization conditions applied to the activated carbons.

### Characterization

The morphology of the precursor materials, the reference carbon, and the activated carbons were studied using scanning electron microscopy (SEM) with a Hitachi S-3400N low-vacuum scanning electron microscope. Samples were dispersed on an aluminum sample holder using carbon tape. Images were collected without previous metallization.

The chemical composition of printed paper waste was determined using an S8 Tiger (Bruker) X-ray fluorescence (XRF) spectrometer, operating in a vacuum atmosphere, a rhodium (Rh) source, a Si(Li) semiconductor detector, and a 10-mm collimator. Measurements were performed using tube voltages of 50 kV for elements Ti–U and 15 kV for Na–Sc, with maximum operating energies of 40 and 4.4 keV, respectively. Analyses were done using pellets of the printed paper supported on boric acid pressed at 5.0 N, and a semiquantitative method (internal calibration) was used to estimate the elements’ concentration.

Fourier transformed infrared (FTIR) spectroscopy was performed at room temperature (25 °C) using a Perkin–Elmer Spectrum 400 spectrometer to characterize the chemical composition of the synthesized carbons, providing information about different chemical bonds and the detection of functional groups. Spectra were collected using 0.1% KBr pellets. The thermal degradation of the precursor material and the synthesized carbons was studied using thermogravimetry (TGA) under a nitrogen and synthetic air flow rate of 50 mL min^−1^ with a Shimadzu DTG60H thermobalance (accuracy ± 2 °C and 0.001 mg), employing a heating rate of 10 °C min^−1^, ranging from room temperature to 900 °C. The crystalline structure of the materials was analyzed using X-ray diffraction patterns collected on a Shimadzu model XRD-6000 diffractometer, with a graphite monochromator, operating with CuKα (λ = 0.15406 nm) radiation generated at 40.0 kV and 30.0 mA, ranging from 10 to 80° 2θ at a scan rate of 2° min^−1^.

Raman spectra were collected in the range of 400–3000 cm^−1^ with an XY Raman Spectrometer (Jasco 5100), using a 2.8 mW argon laser (532 nm, Spectra Physics), and the analyzed region was directly visualized through a 20× microscope objective. The Raman light was collected using a backscattering configuration. The incident light was consistently polarized perpendicularly to the plane of incidence to increase the intensity of the scattered signal, with the data acquired in reflection mode. Powder samples were placed on a pressed slide directly on the microscope stage, with the specific area for spectral recording selected via optical alignment. Three Raman spectra were recorded for each sample.

The N_2_ physisorption isotherm was collected at −196°C using an equipment Micromeritics ASAP 2020. Regarding sample preparation, the degassing conditions were meticulously controlled at a temperature of 200 °C for 360 min under a nitrogen atmosphere, ensuring the ideal preparation of the samples for the adsorption tests. Textural properties were calculated by treating the isotherms using the BET, Dubinin-Astakhov, and the non-local density functional theory (NLDFT) methods.

#### CO_2_ adsorption and regeneration tests

**Fig. 1:**
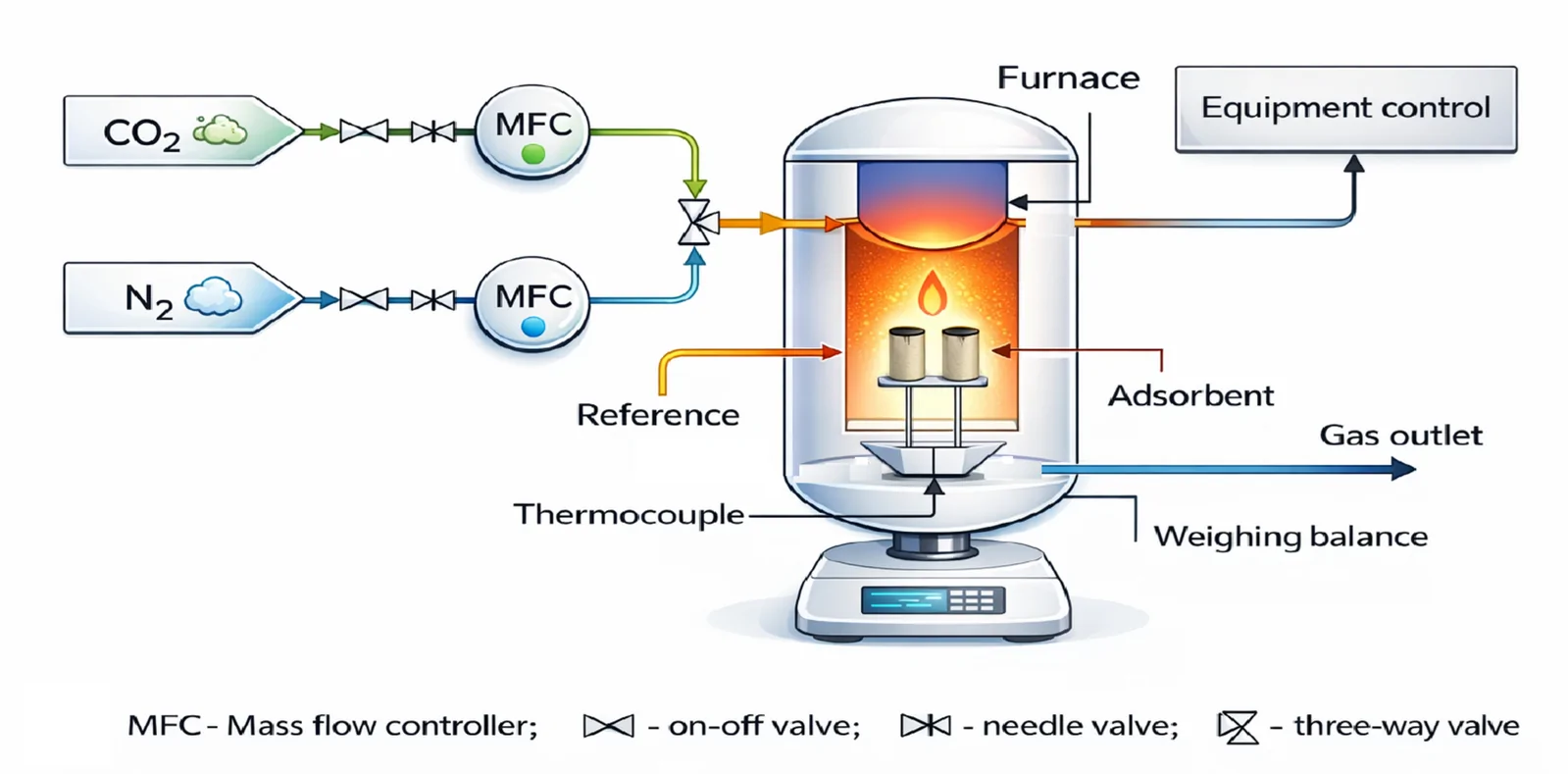
Experimental setup for CO_2_ adsorption by TSA in the TG/DTA equipment (Image created by the authors and improved with AI image generator)

The adsorption characteristics were evaluated using a Shimadzu DTG-60H thermogravimetric analyzer (TG) with a precision of ± 2 °C and 0.001 mg, for CO_2_ adsorption at 30 °C and atmospheric pressure (1 bar), using the experimental setup shown in Fig. 1. Sample pretreatment involved heating to 150 °C for 30 min under N_2_ flow (100 mL min^−1^) to remove gaseous impurities and moisture, followed by cooling to 30 °C for the CO_2_ adsorption process. Adsorption was conducted by purging with a CO_2_:N_2_ = 1:1 mixture at a flow rate of 100 mL min^−1^ for 20 min at 30 °C, after the system reached thermal equilibrium. Desorption was performed by switching the gas flow to N_2_ (100 mL min^−1^) maintained at 150 °C for 15 min. The best-performing adsorbent was selected to investigate the effect of cyclic capture on its adsorption capacity. In cyclic capture experiments, the chosen activated carbon sample was subjected to a degassing step at 150 °C for 30 min before each adsorption test.

The adsorption capacity (*Q*, in mmol g^−1^) of the synthesized activated carbons was calculated using Eq. 1 (Menezes et al. 2025):

1 $$Q=\left[\frac{{m}_{\mathrm{ads}.\mathrm{f}}-{m}_{\mathrm{ads}.\mathrm{i}}}{{m}_{\mathrm{ads}.\mathrm{i}}.M}\right]$$

In which, *m*_ads.f_ represents the final mass after CO_2_ adsorption; *m*_ads.i_ is the initial mass immediately before CO_2_ adsorption, both obtained from the mass gain curves of the materials and expressed in mg; and *M* is the molar mass of CO_2_, expressed as 0.044 g mmol^−1^.

## Results and discussions

### Characterization of the synthesized materials

**Fig. 2:**
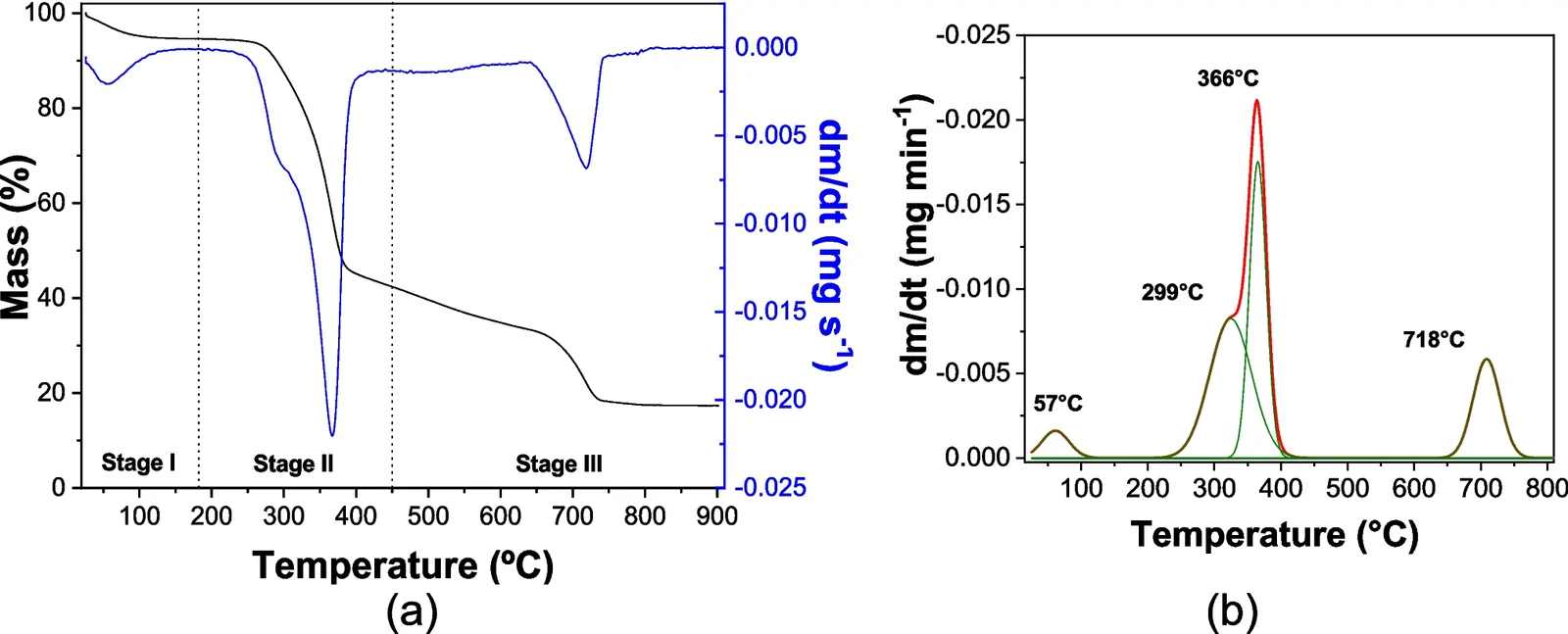
Thermogravimetric analysis of paper waste: **(a)** TG/DTG curves and **(b)** DTG deconvolution (red line - simulated curve; green lines - signals used in deconvolution). Conditions: mass = 8 mg; heating rate = 10 °C min^−1^; nitrogen flow = 50 mL min^−1^

The thermal degradation profile and the optimized temperature range for the pyrolysis process of paper waste were established based on the thermogravimetric analysis data (Fig. 2). Figure 2(a) illustrates the correlation between mass (TG) and its derivative curve (DTG) of the constituent biomass in paper waste. The TG curve exhibits a progressive mass reduction occurring in three distinct stages, which can be attributed to the heterogeneous thermal stability of the various components present in the waste. The deconvolution of DTG is shown in Fig. 2(b).

Figure 2(a) indicates that stage I, occurring at temperatures below 125 °C, can be associated with the evaporation of moisture and volatile compounds. The subsequent degradation stages, observed around 299 and 366 °C in the deconvoluted curve, Fig. 2(b), are attributed to the decomposition of the main organic constituents (hemicellulose and cellulose), leading to a resulting mass loss of approximately 50%. The final degradation event, occurring at 718 °C, is ascribed to the thermal decomposition of inorganic fillers, primarily calcium carbonate, commonly found in paper waste (Bhardwaj et al. 2023).

The weight loss stabilizes significantly above 750 °C, reaching a constant plateau. Therefore, the thermogravimetric analysis suggests that a pyrolysis temperature of 800 °C is optimal for ensuring the complete carbonization of paper waste and the removal of residual volatile matter. This temperature ensures the formation of a thermally stable carbonaceous matrix, providing a consistent precursor for the subsequent activation stages.

**Fig. 3:**
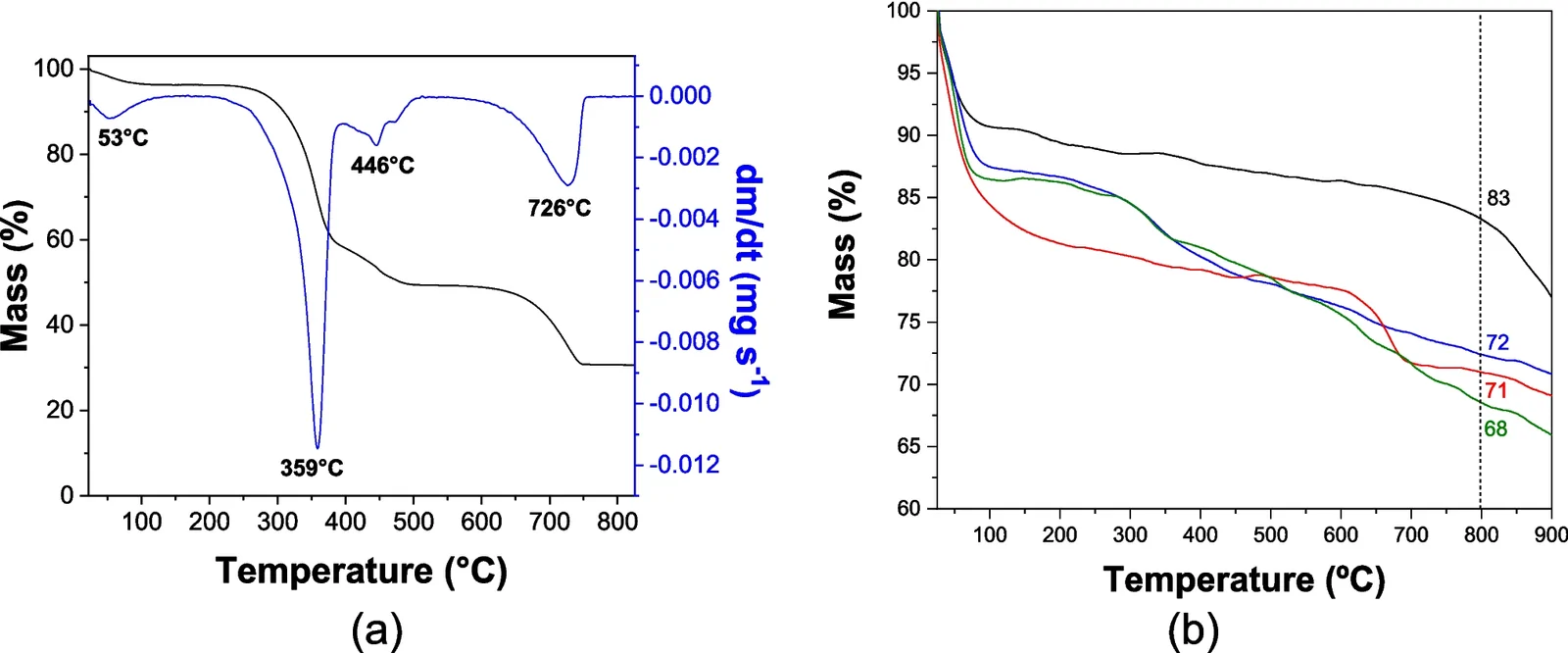
**(a)** Thermogravimetric analysis of paper waste under synthetic air flow (50 mL min^−1^); **(b) **Thermogravimetric curves of the carbons synthesized from printed paper waste. Conditions: sample mass ≈ 8 mg, heating rate = 10 °C min^−1^, and nitrogen flow = 50 mL min^−1^

From the thermogravimetric curve of paper waste under an oxidizing atmosphere (synthetic air), shown in Fig. 3, it was observed an initial mass loss due to moisture and volatile compounds, as also shown in Fig. 2. In the range of 250–550°C, the macrocomponents (hemicellulose and cellulose) are burned in the oxidative atmosphere in a complex mechanism, releasing CO and CO_2_. The mass loss centered at 726 °C can be ascribed to the thermal decomposition of CaCO_3_ and the residue is associated with the ash content of the sample, corresponding to about 30%.

The thermogravimetric curves of the non-activated and activated carbons are shown in Fig. 3(b). The profiles demonstrated similar behavior in the degradation of the activated carbons AC-KOH, AC-KCO, and AC-KHP. In contrast, the non-activated carbon (RC-PP), obtained from the carbonization of W-PP at 800 °C, exhibited greater thermal stability, with a mass loss of only 18%, lower than that observed for the activated carbons. However, the activated carbons still exhibit a high carbon yield between 68% and 72%.

Mass loss in activated carbons was attributed to the slow degradation of the carbon matrix, which is resistant to the activation process, and these events were related to the development of the porous structure (Fiuza-Junior et al. 2016). However, it is important to note that some residues from the activating agents might be present in the final material, interfering with the shape of the TG curves in Fig. 3(b).

Table 1 presents the results of the X-ray fluorescence (XRF) analysis. Based on these results, it is observed that paper waste exhibits a high content of calcium oxide, which is mainly associated with the filler materials used in its manufacture, such as calcium carbonate and kaolin. These components are directly related to the high ash contents obtained after the carbonization of this material (Vukoje and Rožić 2018). Furthermore, the presence of metals such as Al, Ca, Cr, Cu, Pb, Sn, Ti, and Na in the form of oxides in printed paper waste can be attributed to the inks and coatings used in printing processes. In particular, the detection of aluminum may also indicate contributions from materials present in the composite of W-PP structures, such as plastic and aluminum layers. The presence of inks in printed paper waste further supports this interpretation.

**Table 1:** XRF analysis of paper waste used as precursor material

Component	CaO	SiO_2_	Cl	MgO	Al_2_O_3_	Fe_2_O_3_	SO_3_	TiO_2_
Concentration (%)	93.33	1.91	1.20	0.87	0.70	0.55	0.47	0.35
Component	**Na**_**2**_**O**	**P**_**2**_**O**_**5**_	**SrO**	**K**_**2**_**O**	**Cr**_**2**_**O**_**3**_	**CuO**	**ZnO**	-
Concentration (%)	0.29	0.11	0.09	0.05	0.05	0.02	0.02	-

**Fig. 4:**
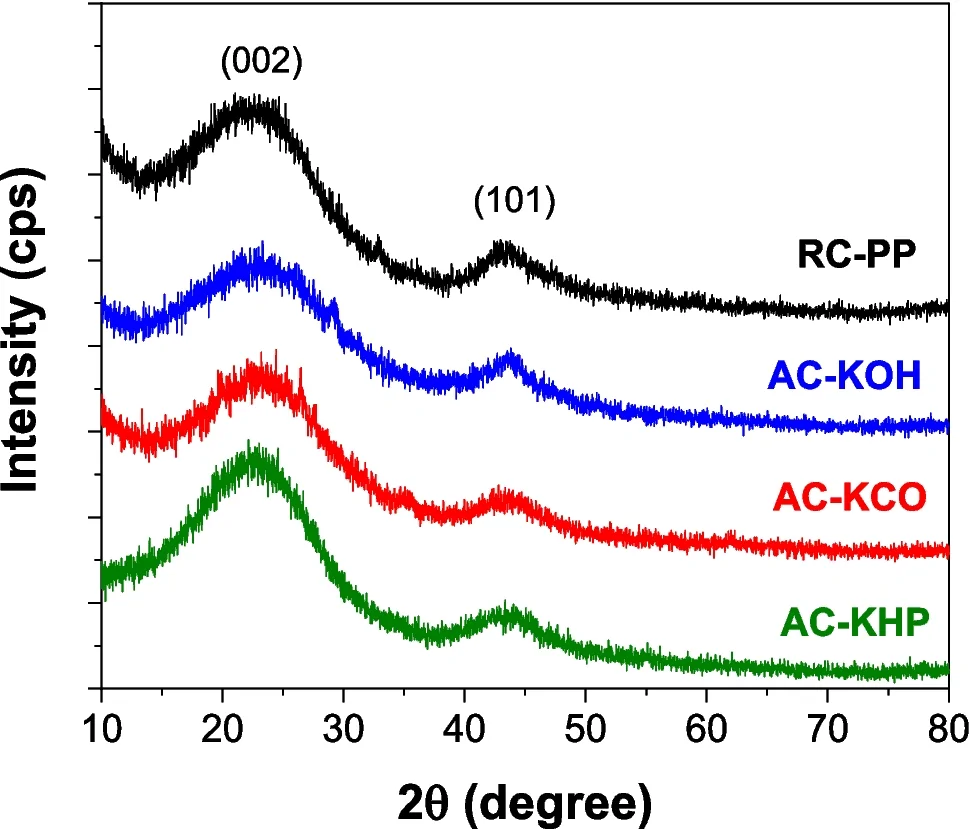
X-ray diffraction patterns of the synthesized carbons

Figure 4 illustrates the XRD patterns, in which it is observed that the activated carbons and the reference carbon exhibit two broad and relatively low-intensity peaks at 2*θ* approximately at 23.2° and 43.5°. These peaks are characteristic of the (002) and (101) reflections of partially graphitic domains embedded in a predominantly amorphous carbon matrix, evidencing low crystallinity in the carbon network, with an intermediate organization between graphite and amorphous carbon (Puthusseri et al. 2014; Fiuza-Junior et al. 2016). The shift in the diffraction peak (002), from 26.3° in graphite to 23.2° in the synthesized carbons, suggests an increase in the interlayer spacing in the carbons.

**Fig. 5:**
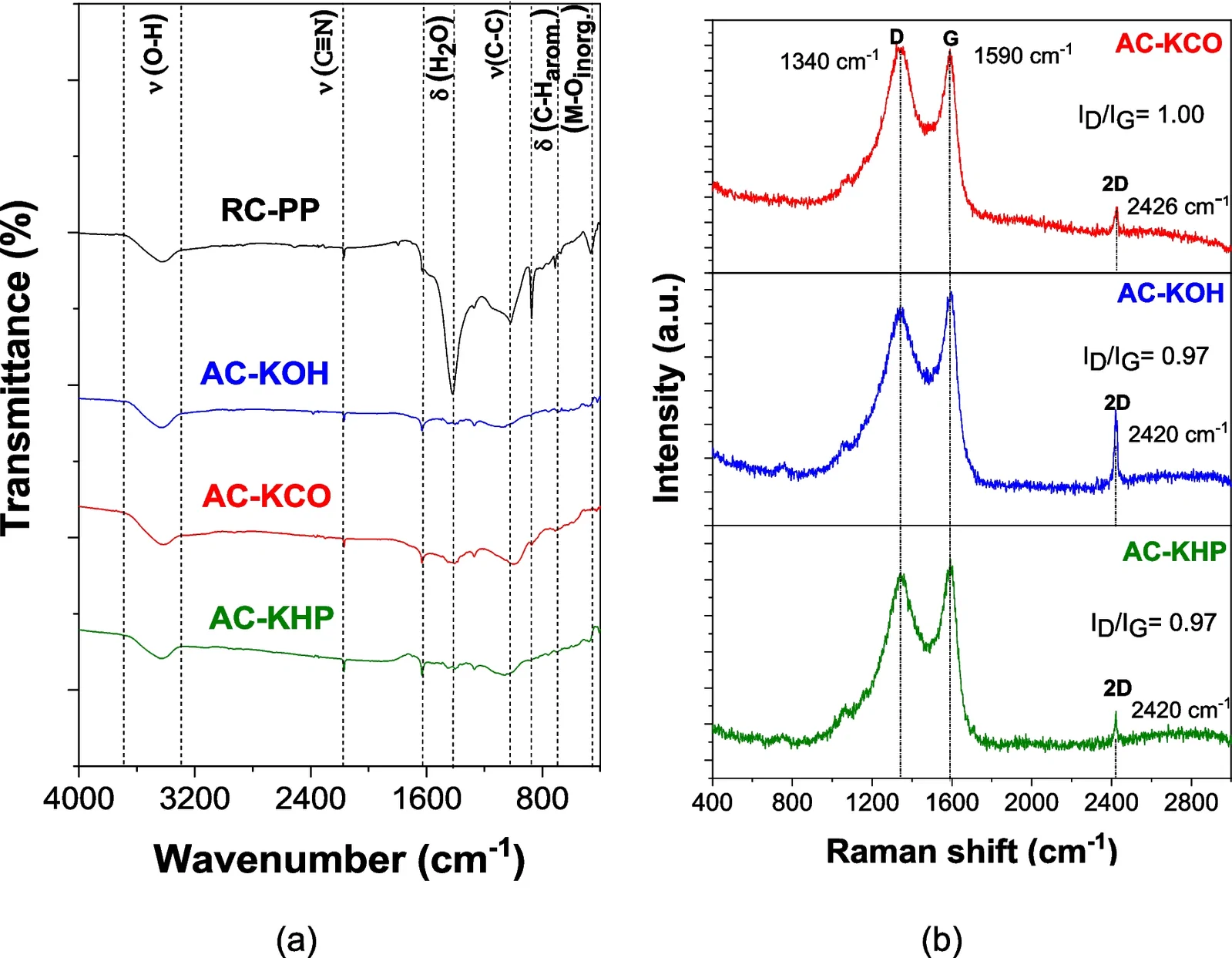
**(a)** FTIR spectra of the activated carbons; **(b)** Raman spectra of the activated carbons

Figure 5(a) presents the FTIR spectra of the activated carbons AC-KOH, AC-KCO, and AC-KHP, along with the spectrum of the reference carbon RC-PP, and Fig. 5(b) shows the Raman spectra of the activated carbons.

The analysis of the FTIR spectrum, Fig. 5(a) reveals the presence of OH functional groups linked by hydrogen bonds in the 3500–3300 cm^−1^ band. The low-intensity absorption band at 2200 cm^−1^ can be attributed to the stretching vibration of the C≡N bond, likely associated with nitrogen-containing surface groups originating from ink residues or additives present in the printed paper precursor, which underwent transformation during the carbonization process. The bands in the 1700–1550 cm^−1 ^and 1500–1250 cm^−1^ regions are associated with the angular deformation of the H–O–H bond and C–H bond from residual aliphatic groups, respectively. The band in the frequency range of 1024 cm^−1^, corresponding to C–C groups, is related to the stretching vibration of the carbon skeleton within aromatic or disordered aliphatic structures, commonly found in carbonaceous materials derived from the thermal decomposition of cellulose and lignin present in printed paper waste. The well-defined absorption bands observed at approximately 881 and 697 cm^−1^ are attributed to the in-plane and out-of-plane bending vibrations of C–H bonds in aromatic rings, respectively, characteristic of the reference carbon sample RC-PP. Additionally, the band at 449 cm^−1^ is associated with M–O stretching vibrations, indicating the presence of inorganic metal–oxygen species likely originating from residual inorganic components in the precursor or from the activation process. Similar results were found by Zu et al. (2013) and Pirzadeh and Ghoreyshi (2014) in studies of carbons derived from paper waste and paper pulp subjected to pretreatment.

The vibrational modes in the three samples, as studied from the Raman spectra and analyzed in the range of 300–3200 cm^−1^, are shown in Fig. 5(b). The peaks at 1340 and 1590 cm^−1^ represent the D and G bands, respectively (Liu et al. 2019; Sundriyal et al. 2021). The G band appears due to in-plane vibrations of sp^2^ hybridized carbons, representing the graphitic order, while the D band originates from defects and disordered graphite present in the amorphous carbon. The *I*_D_/*I*_G_ ratio for the AC-KCO sample was recorded as 1.00, indicating a balanced relationship between the degree of graphitization and disorder. Meanwhile, for the AC-KOH and AC-KHP samples, the ratio was recorded as 0.97, suggesting a slightly higher degree of graphitization in relation to the disordered phase.

The 2D band, in the range of 2420–2430 cm^−1^, corresponds to a graphitic lattice mode with E_2g_ symmetry (Al-Jishi et al. 1982; Dresselhaus et al. 2002; Sadezky et al. 2005). The presence of these bands is due to phonon processes that are only possible in graphitic or sheet-like carbons. Phonons are collective excitations that represent the vibrational motion of atoms within a crystal lattice and play a significant role in determining the thermal and electrical properties of solids. This phenomenon has also been observed by other authors (Chen et al. 2017; Singh et al. 2021). It is observed that the AC-KOH carbon shows a significant increase in the 2D band compared to the other carbons. This suggests a turbostratic carbon structure arrangement of non-graphitic carbon related in depth to the abundant composition of sp^2^ hybridized carbon in a stack of nanometer-sized graphene layers with disordered structural characteristics (Ruhaimi et al. 2024).

**Fig. 6:**
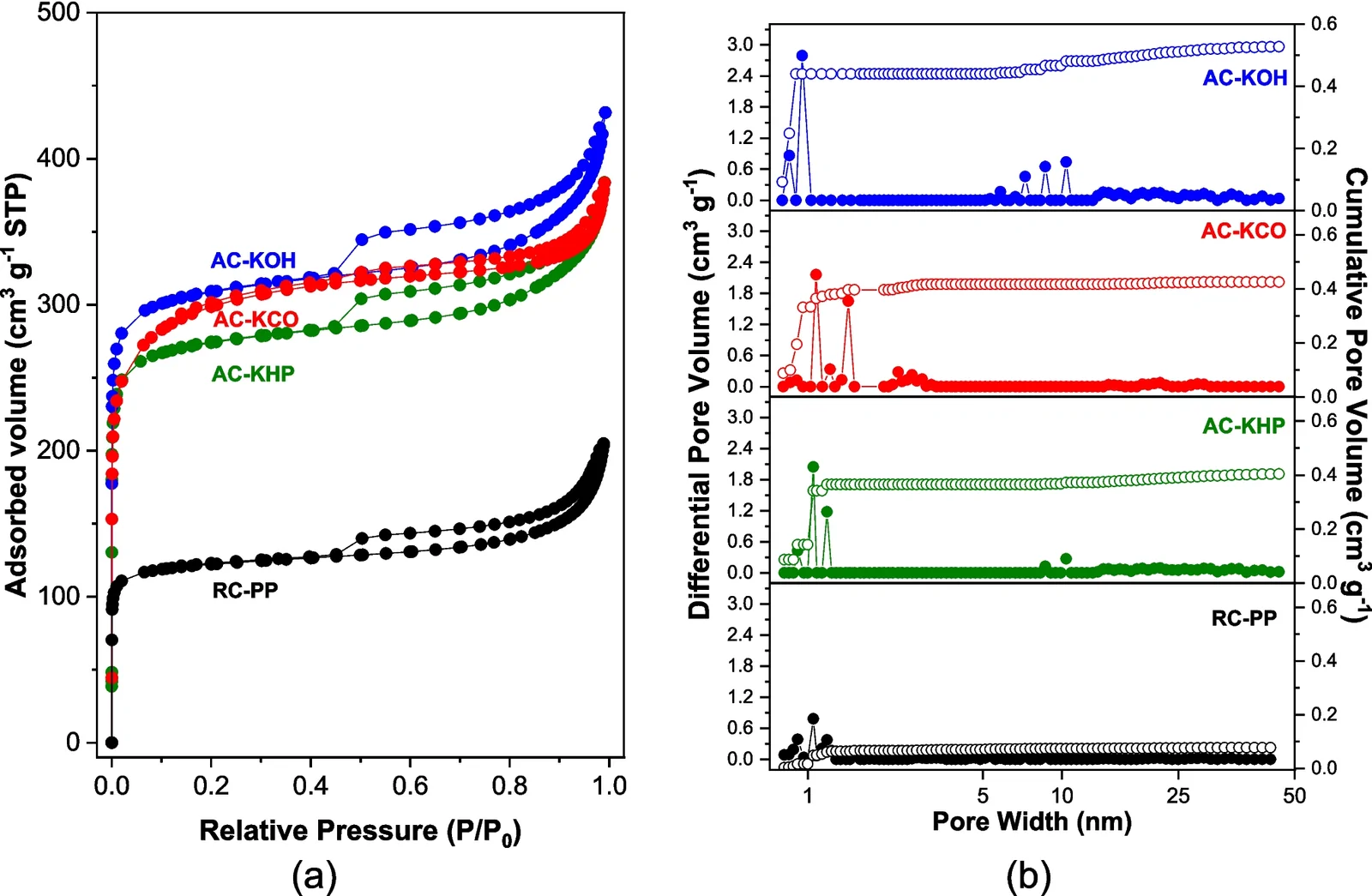
**(a)** N_2_ adsorption–desorption isotherms of the activated carbons and the reference carbon. **(b)** Pore size distribution calculated by the NLDFT method

Nitrogen physisorption isotherms and NLDFT pore size distribution are shown in Fig. 6(a) and 6(b), respectively. As illustrated in Fig. 6(a), the activated carbons and the reference carbon exhibited Type I/IV hybrid adsorption isotherms, typical of hierarchical pore distribution, i.e., ranging from micropores to mesopores region. These isotherms are characterized by a sharp curvature towards the relative pressure axis (*P*/*P*₀), followed by a plateau indicative of saturation, a behavior typical of the Langmuir isotherm (Xu et al. 2024). It is noteworthy that, at low relative pressures (*P*/*P*₀ < 0.02), the N₂ adsorption showed a rapid increase (Li et al. 2019). However, at relative pressures above *P*/*P*_0_ > 0.4, one could observe the presence of hysteresis loops, characteristic of capillary condensation on mesopores (Thommes et al. 2015).

These hybrid isotherms were confirmed by the presence of various pore sizes in the activated carbon, including micropores and mesopores, as shown in the NLDFT pore size distribution, Fig. 6(b). The textural properties determined by BET, DA, and NLDFT methods are shown in Table 2.

**Table 2:** Textural properties of the prepared adsorbents

Sample	^a^*S*_BET_ (m^2^ g^−1^)	^b^*S*_mic_ (m^2^ g^−1^)	^c^*V*_T_ (cm^3^ g^−1^)	Micropore regions			^d^*V*_micro_ (cm^3^ g^−1^)	^e^*V*_meso_	Vmicro/Vtotal
				**0.7–1.0(nm)**	**1.0–1.5(nm)**	**1.5–2.0(nm)﻿**		**2.0–50(nm)**	
RC-PP	432	518	0.077	0.016	0.047	0.003	0.066	0.011	0.86
AC-KOH	1091	1329	0.528	0.440	-	-	0.440	0.088	0.83
AC-KCO	1059	1310	0.425	0.333	0.063	-	0.396	0.029	0.93
AC-KHP	969	1139	0.404	0.345	0.020	-	0.365	0.039	0.90

^a^Specific surface area

^b^Micropore surface area calculated by the Dubinin–Astakhov method

^c^Total pore volume estimated by NLDFT method

^d^Micropore volume estimated by NLDFT

^e^Mesopore volume estimated by NLDFT

Based on the data presented in Table 2, chemical activation significantly increases the BET specific surface area of activated carbons compared to the reference carbon (RC-PP). The AC-KOH sample showed a surface area of 1091 m^2^ g^−1^, which is approximately 2.5 times higher than that of RC-PP (432 m^2^ g^−1^). Similarly, the AC-KCO (1059 m^2^ g^−1^) and AC-KHP (969 m^2^ g^−1^) samples also exhibited BET-specific surface areas more than twice as large as that of the reference carbon. This increase can be attributed to the oxidation of the carbonaceous structure promoted by potassium at temperatures above 700 °C (Fiuza-Junior et al. 2016), as well as the gases released during the thermal decomposition of the activating agents. The release of these gases during carbonization promotes an increase in the BET-specific surface area and in the pore volume of the activated carbons (Xu et al. 2024). These results clearly reveal a notable variation in textural properties when compared to the reference carbon (RC-PP), thus demonstrating the activating potential of KOH, K_2_CO_3_, and KHC_8_H_4_O_4_ in the single-step activation process of pretreated paper waste.

A further analysis of the action of activating agents KOH, K_2_CO_3_, and KC_8_H_4_O_4_ based on textural properties presented in Table 2 reveals that all were highly effective in creating and expanding the micropore network in activated carbons. The substantial increase in the micropore surface area, calculated by the Dubinin–Astakhov method, evidences this effect: AC-KOH (1329 m^2^ g^−1^), AC-KCO (1310 m^2^ g^−1^), and AC-KHP (1139 m^2^ g^−1^), values more than twice that of the reference carbon RC-PP (518 m^2^ g^−1^).

These results suggest that the chemical activation process promoted the development of an extensive network of ultrafine pores. Potassium hydroxide (KOH) is known for its high reactivity and proved to be particularly efficient in generating the largest micropore surface area among the three agents studied. Although the values obtained for AC-KCO and AC-KHP are slightly lower than that of AC-KOH, they still represent a significant increase compared to the reference carbon, also confirming the effectiveness of K_2_CO_3_ and KC_8_H_4_O_4_ in generating micropores. The observed variations between the activated materials can be attributed to differences in the thermal decomposition mechanisms of each agent and the release of gases that contribute to porosity development.

Jinyang et al. (2024) propose that this performance may be related to the formation of a higher proportion of micropores or the creation of more accessible and effective structures for adsorption. To confirm which hypothesis is correct, the nonlocal density functional theory approach was used, as recommended by IUPAC (Thommes et al. 2015).

The pore size distribution was determined using the NLDFT method, applying the slit pore model, which allowed for a quantitative assessment of the presence of micropores (diameter < 2 nm) and mesopores (2 nm < diameter < 50 nm). According to the classification proposed by IUPAC, micropores can be further subdivided into ultramicropores (< 0.7 nm), supermicropores (0.7–1.0 nm), and micropores (1.0–2.0 nm) (Cordeiro et al. 2024). The results presented in Table 2 indicate that the AC-KOH sample is predominantly supermicroporous, with most pores ranging from 0.7 to 1.0 nm, accounting for 83.3% of the total pore volume. Additionally, the presence of narrow mesopores, ranging from 5.8 to 13.0 nm (16.7%), was also present. These findings suggest that AC-KOH exhibits a hierarchical porous structure, a desirable characteristic for materials used in adsorption and catalysis applications.

In the activated carbons AC-KCO and AC-KHP, the micropores are also predominantly distributed in the 0.7 to 1.5 nm range, as shown in Table 2. For AC-KCO, supermicropores account for 78.4% of the total pore volume, while 14.8% fall within the 1.0 to 1.5 nm range, indicating a significant distribution within the broadened microporosity. The activation of printed paper waste treated with potassium carbonate caused strong reactions to the carbonaceous structures, inducing carbon oxidation to CO_2_ and breaking down structures, thus promoting microporosity with a small amount of mesopores. In comparison, AC-KHP, which has a smaller total pore volume, has the largest distribution of micropores in the supermicropore range (85.4%) with a significant contribution in the 1.0–1.5 nm range (4.95%).

**Fig. 7:**
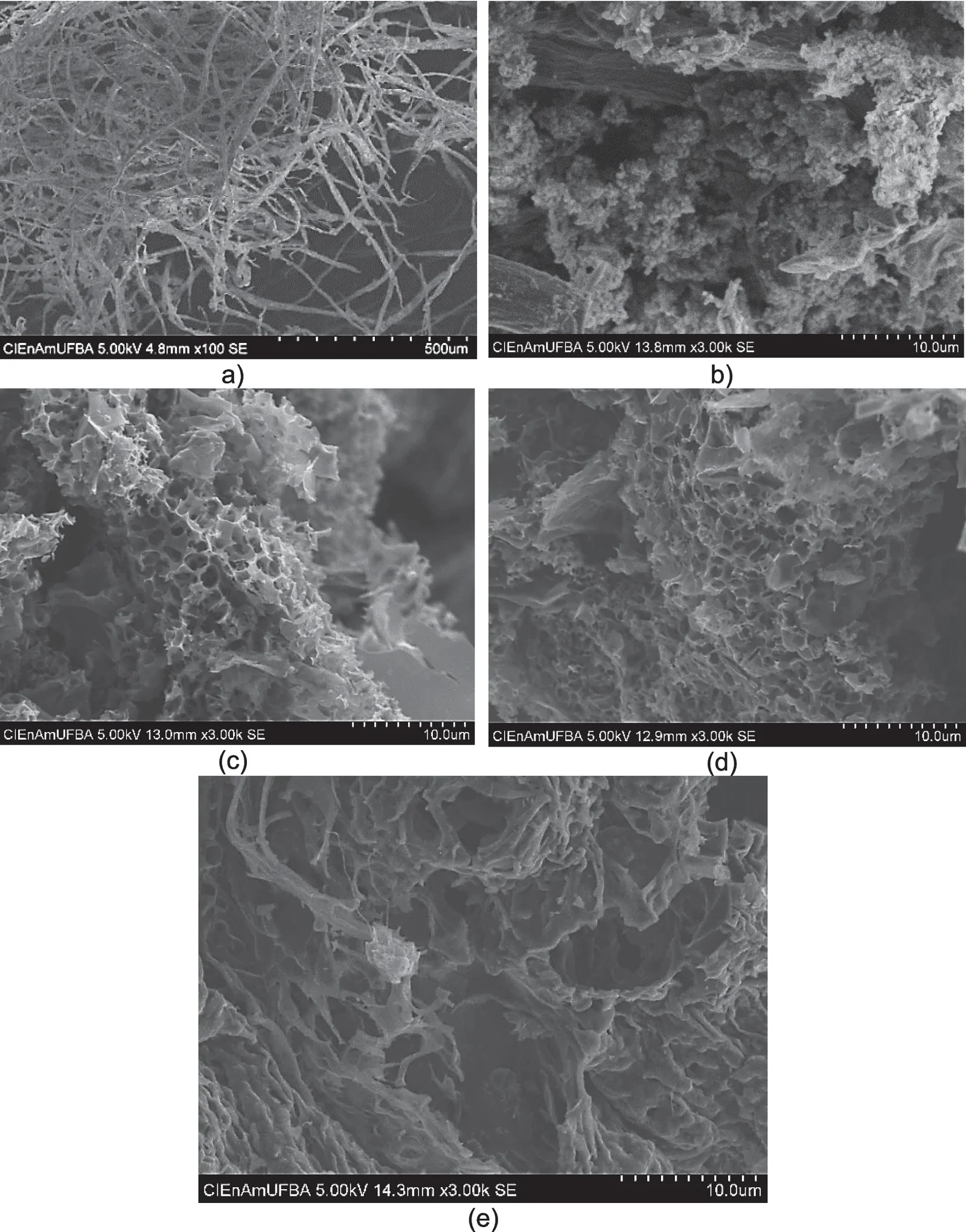
Scanning electron microscopy images: (**a**) shredded printed paper waste, (**b**) reference carbon RC-PP, (**c**) activated carbon AC-KOH, (**d**) activated carbon AC-KCO, and (**e**) Activated carbon AC-KHP

SEM analysis was performed to examine the morphology of the paper waste and the different carbons obtained from it, illustrated in Fig. 7(a)–7(e). These images provide information about structural transformations during the carbonization process.

All SEM images in Fig. 7 were acquired at consistent magnifications: 100 × for the reference carbon (RC-PP), with a scale bar of 500 µm, and 3000 × for all other samples, with a scale bar of 10 µm. This allows a direct visual comparison of morphological transformations.

As illustrated in Fig. 7(a), the morphology of shredded paper waste is characterized by agglomerates of fibers with a rough surface (sometimes described in the literature as scaly ribbons). These fibers are devoid of pores or cavities, which is consistent with the high concentration of cellulose and hemicellulose in their composition (Vukoje and Rožić 2018).

Figure 7(b) shows that the reference carbon (RC-PP), produced at 800 °C without activation, exhibits a rough texture with multiple cavities, still preserving several carbonized ribbon-shaped fibers that reflect the morphology of the original material. However, this structure appears dense and collapsed in comparison to the activated samples, suggesting a limited contribution to overall porosity and, consequently, a low surface area and adsorption capacity, which necessitates subsequent chemical activation.

The activation with potassium hydroxide (AC-KOH), as demonstrated in Fig. 7(c), leads to the formation of a structure with significant roughness and numerous cavities, indicating the presence of microporosity, not detectable by N_2_ physisorption analysis.

Visually, the AC-KOH surface exhibits the most severe and extensive erosion among all samples, resulting in an extremely heterogeneous texture. This highly aggressive etching is a direct manifestation of the redox reaction between carbon and KOH.

According to Heidarinejad et al. (2020), the genesis of these cavities in the KOH-activated carbon is attributed to the redox reaction between the carbon and KOH, as well as gasification with the release of H_2_, H_2_O, CO, and CO_2_. During the activation process, the generation of K^+^ and K_2_CO_3_ within the carbonaceous matrix contributes to stability and increases the interlayer spacing, enhancing the efficiency and adsorption capacity of the activated carbon. Furthermore, metallic potassium, originating from KOH during pyrolysis, intercalates into the carbon structure and reacts with the carbon walls.

Figure 7(d) illustrates the AC-KCO sample, whose porosity developed during activation with K_2_CO_3_. The obtained material is a result of its decomposition at high temperatures under an inert atmosphere to produce K_2_O and CO_2_ and subsequent reduction of K_2_O by carbon, producing elemental potassium (K) and carbon monoxide (CO) (Lehman et al. 1998). It is important to consider the possibility of reduction of K_2_O by CO at higher temperatures resulting in elemental potassium and CO_2_. The highly permeable elemental potassium penetrates the carbon structure, triggering the formation of micropores. Concomitantly, the gases released during the process promote pore expansion (Adinata et al. 2007; Foo and Hameed, 2012). While also highly porous, the AC-KCO morphology appears to feature more defined and potentially larger cavity structures compared to the general roughness seen in AC-KOH. The visual distinction supports the literature suggesting that the decomposition of potassium carbonate generates cavities on the surface of the activated carbon shaped by the locations occupied by the activating agent. These empty spaces act as channels, connecting the micropores and mesopores formed in the activated carbon (Abbas and Ahmed, 2016).

The porosity development and surface roughening observed in the AC-KHP sample, Fig. 7(e) shows clear evidence of activation, presenting an intermediary level of surface destruction between the RC-PP and the highly etched AC-KOH. This morphology is attributed to the influence of potassium species (K_2_CO_3_, K_2_O, and K) formed during the thermal decomposition of KC_8_H_4_O_4_ under a nitrogen atmosphere. The infiltration of these species into the carbonaceous framework during activation leads to the creation of micropores and mesopores, as evidenced by the textural analysis, Fig. 6(a) and 6(b).

Considering that the potassium hydrogen phthalate (KHC_8_H_4_O_4_) was used in an aqueous solution to impregnate the printed paper prior to the pyrolysis stage, the chemical equilibrium can produce low concentrations of phthalate anions (C_8_H_4_O_4_^2−^) and oxonium (H_3_O^+^), since hydrogen phthalate is weakly acidic, pKa = 5.41 (Gagliardi et al. 2010). The subsequent thermal decomposition of KHC_8_H_4_O_4_ in N_2_ results in K_2_CO_3_ and char (Belcher et al. 1960; Newkirk and Laware, 1962). Afterward, K_2_CO_3_ decomposes into K_2_O and CO_2_ and reacts as discussed above.

### Study of the temperature swing adsorption process of CO_2_

**Fig. 8:**
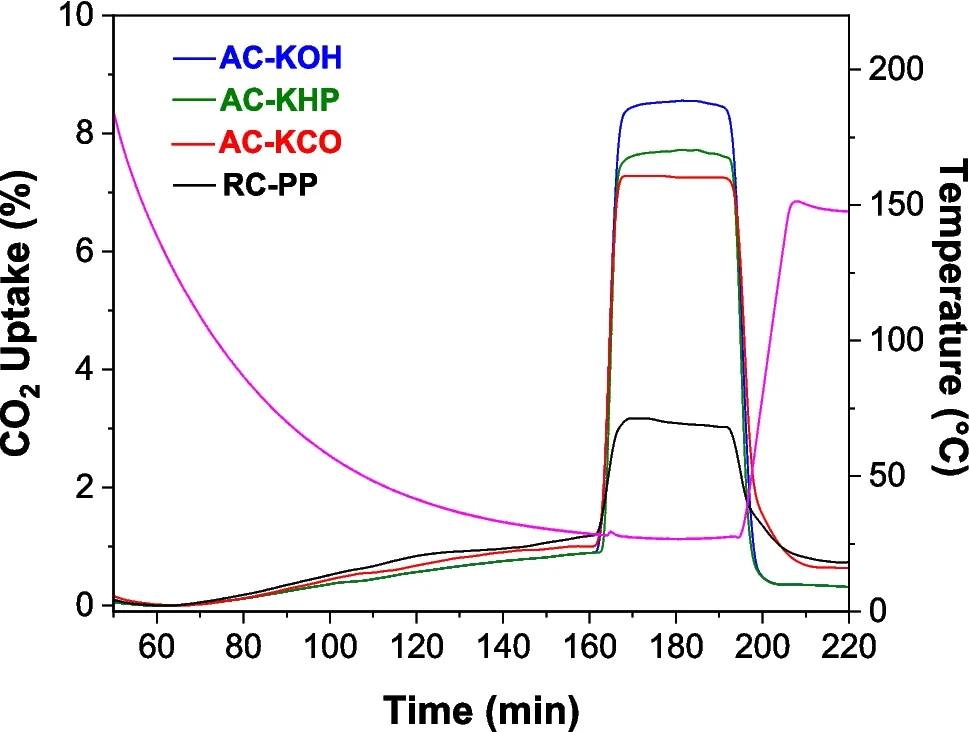
CO_2_ adsorption–desorption curves on carbons prepared from printed paper waste determined by thermogravimetry (*T* = 30 °C; *P* = 1 bar; mass = 8 mg; *β* = 10 °C·min^−1^, *F* = 100 mL·min.^−1^, 50% CO_2_ + 50% N_2_)

The CO_2_ adsorption–desorption curves of the activated carbons derived from printed paper waste, measured at 30 °C and 1 bar, are shown in Fig. 8.

A considerable increase in CO_2_ adsorption is observed for the chemically activated carbons (AC-KOH, AC-KCO, and AC-KHP) compared to the nonactivated carbon (RC-PP). The activated carbon AC-KOH (7.64%) exhibited the highest CO_2_ adsorption capacity, a result probably due to the improved textural properties achieved by KOH activation. When compared to AC-KCO and AC-KHP, AC-KOH showed the highest BET surface area (1091 m^2^ g^−1^) and, significantly, the largest DA micropore area (1329 m^2^ g^−1^) and NLDFT micropore volume (0.440 cm^3^ g^−1^), features that enhance CO_2_ physisorption. Furthermore, the enhanced basicity imparted by KOH explains the selective CO_2_ adsorption.

**Fig. 9:**
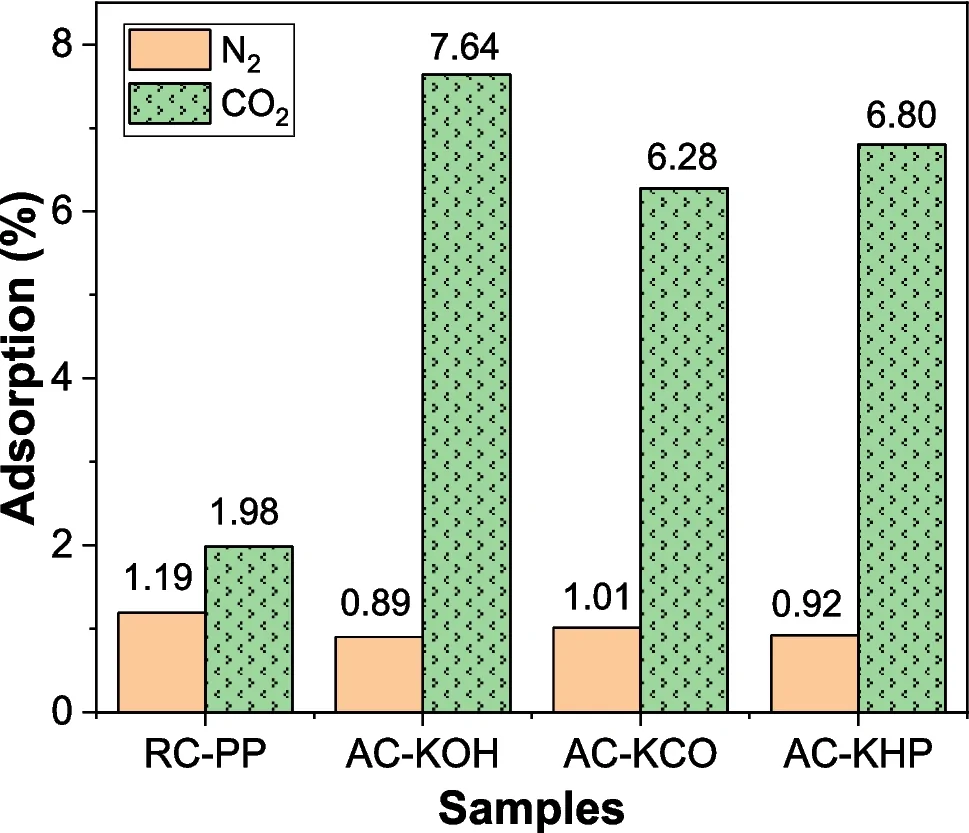
Quantity adsorbed of N_2_ and CO_2_ for the reference carbon and the activated carbon

The comparison of CO_2_ and N_2_ adsorption capacities between the reference carbon and the activated carbons is shown in Fig. 9.

The comparative analysis of CO_2_ capture results (Fig. 9) reveals that, although AC-KCO (6.28%, 1.93 mmol g^−1^) possesses a larger specific surface area (*S*_BET_) and micropore volume, its lower percentage of micropores in the 0.7–1.0 nm range may be a determining factor in its inferior performance compared to AC-KHP (6.80%, 2.03 mmol g^−1^), which exhibits a slightly greater micropore volume in this range (*V*_micro_ [0.7–1.0 nm] = 0.345 cm^3^ g^−1^) in comparison with AC-KCO (*V*_micro_ [0.7–1.0 nm] = 0.333 cm^3^ g^−1^). The best result was obtained for AC-KOH (7.64%, 2.29 mmol g^−1^), which presented the highest micropore volume, exclusively in the supermicropore region.

**Fig. 10:**
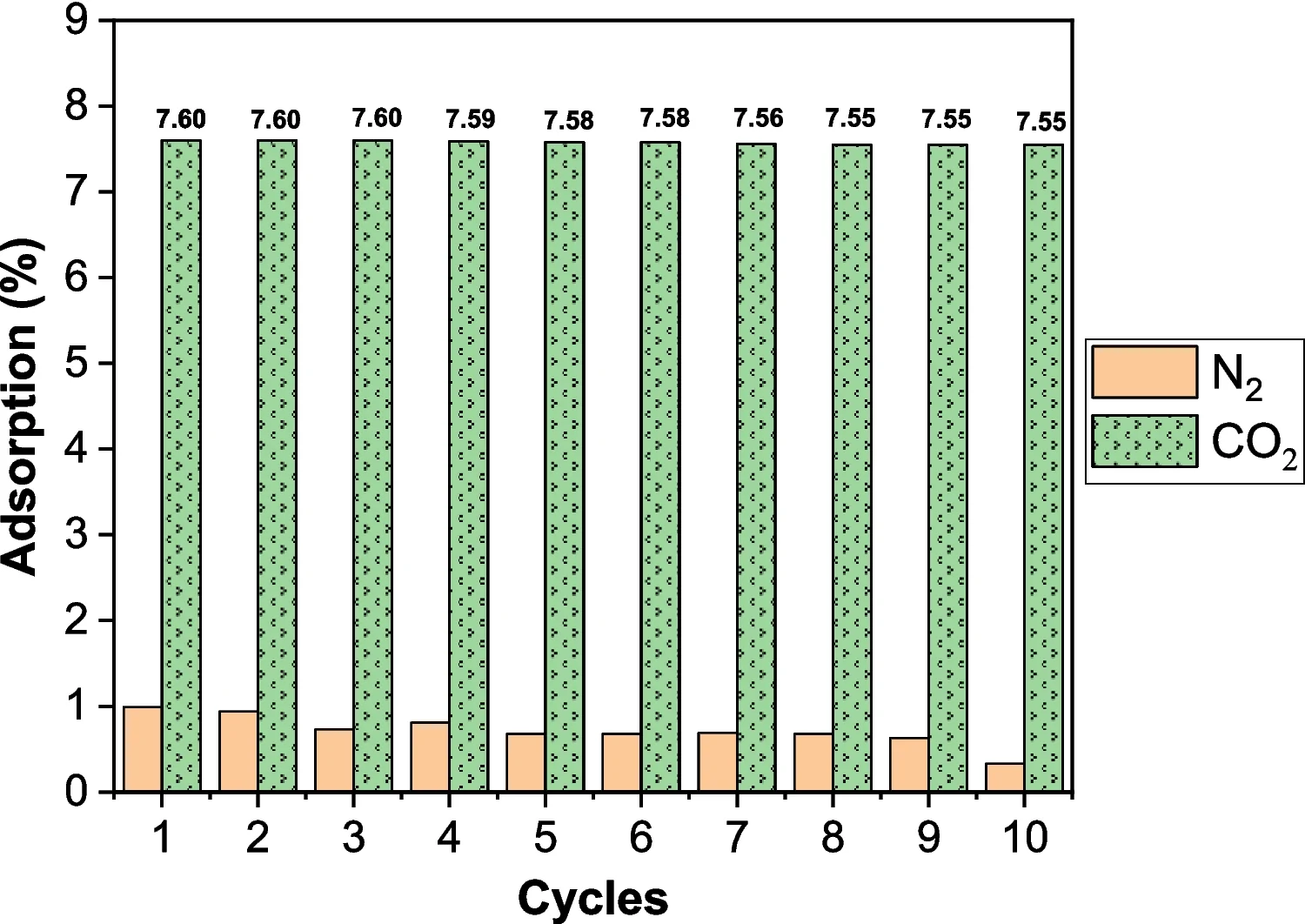
Quantity adsorbed of N_2_ and CO_2_ by activated carbon AC-KOH during 10 adsorption–desorption cycles as determined by TG

Figure 10 shows the process over 10 cycles to evaluate the adsorption–desorption stability of CO_2_ for the best activated carbon.

In the cyclic stability test results of AC-KOH, it can be observed that after ten adsorption–desorption cycles, the CO_2_ adsorption capacity of the activated carbon remains basically unchanged, with an average fluctuation range of less than 1% (Fig. 10), showing good cycle stability and regeneration performance. Furthermore, the amount of CO_2_ adsorbed by AC-KOH was maintained in the range of 7.55%–7.60% (2.27–2.28 mmol g^−1^), with the first cycle value similar to the 7.64% (2.29 mmol g^−1^) obtained in the adsorption–desorption test (Fig. 7), which allows us to verify the high CO_2_ adsorption performance of this activated carbon and its high regeneration capacity over 10 cycles.

**Fig. 11:**
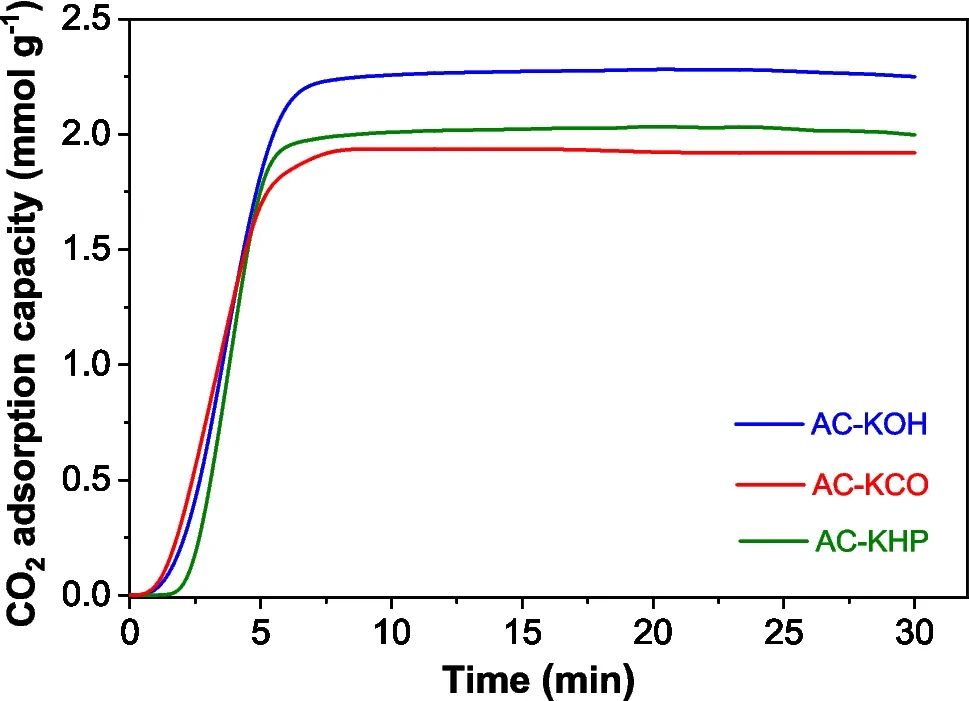
CO_2_ adsorption capacity of the activated carbons as a function of time

The adsorption capacity of CO_2_ for AC-KOH, AC-KCO, and AC-KHP was analyzed at 30 °C and 1 bar, as shown in Fig. 11.

According to Fig. 11, the activated carbons AC-KOH, AC-KCO, and AC-KHP exhibit a rapid initial adsorption rate due to the availability of active sites. However, as adsorption progresses, the occupation of these sites and the saturation of the pores by CO_2_ molecules increase the resistance to diffusion in the carbon adsorbents. Equilibrium is reached in approximately 6 min for the three synthesized carbons, achieving adsorption capacities of 2.29 mmol g^−1^ (AC-KOH), 1.93 mmol g^−1^ (AC-KCO), and 2.03 mmol g^−1^ (AC-KHP).

### Comparison with other ACs obtained from waste

Table 3 compares the performance of the activated carbon adsorbents synthesized in this work with others derived from paper and agroindustrial waste in CO_2_ adsorption via TSA at 30 °C and 1 bar.

**Table 3:** Comparison of the adsorption capacity in the TSA process of activated carbons prepared in this work with other published in literature, obtained from different materials at a temperature of 30 °C and a pressure of 1 bar

Feedstock	Activator:carbon	^a^*T*_CR_ (°C)	^b^*T*_CA_ (°C)	^c^*V*_T_ (cm^3^ g^−1^)	^d^*S*_BET_ (m^2^ g^−1^)	^e^*Q*_CO2_ (mmol g^−1^)	References
	KOH (1:1)	N/A ^f^	800	0.528	1091	2.29	
Printed paper	K_2_CO_3_ (1:1)	N/A ^f^	800	0.425	1059	1.93	This work
	KHC_8_H_4_O_4_ (1:1)	N/A ^f^	800	0.404	969	2.03	
Eucalyptus wood	Two steps H_3_PO_4_/KOH (3.5:1)	450	900	1.275	2595.0	4.10	Heidari et al. (2014)
Persian Ironwood	KOH (3:1)	700	900	0.826	1935.8	3.77	Nowrouzi et al. (2017)
	H_3_PO_4_ (3:1)	600	800	1.585	1904.8	5.05	
Leucaena wood	H_3_PO_4_ (2.5:1)	600	550	1.420	1512.6	3.55	Asadi-Sangachini et al. (2019)
	KOH (1.5:1)	600	900	0.500	727.7	2.84	
Coffee ground	KOH (1:1)	N/A ^f^	600	0.400	256.5	2.00	Rattanaphan et al. (2020)
	KOH (1/1)/C_2_H_8_N_2_	600	500	0.400	11.8	2.48	
Olive pruning residue	KOH (6.38:1)	500	789	1.790	3526	1.89	Ramos et al. (2024)

^a^*T*_CR_: carbonization temperature

^b^*T*_AC_: activation temperature

^c^Total pore volume

^d^BET surface area

^e^*Q*_CO2_: adsorption quantity

^f^N/A: not applicable

The results show that some KOH-activated carbons, such as those obtained by Rattanaphan et al. (2020), Ramos et al. (2024), or Asadi-Sangachini et al. (2019), listed in Table 3, exhibit similar or only slightly better performance than those synthesized in this work, regardless of the number of steps or the activating agent concentration. However, the carbons with the highest CO_2_ adsorption consistently utilize a higher proportion of chemical activating agent than the 1:1 ratio applied in AC-KOH, AC-KCO, and AC-KHP. These results indicate that the activated carbons prepared in one step with a precursor/activating agent ratio of 1:1 (AC-KOH, AC-KCO, and AC-KHP) show acceptable potential, justifying the use of printed paper as a suitable precursor for the preparation of AC for CO_2_ capture.

## Conclusions

This study demonstrates that microporous activated carbons can be effectively manufactured from paper waste via a process involving pretreatment, impregnation, and chemical activation coupled with direct carbonization under an inert atmosphere. This strategy bypasses the separate precarbonization stage, leading to a decrease in energy consumption compared to conventional two-step procedures.

The activation of printed paper waste with KOH, K_2_CO_3_, and KHC_8_H_4_O_4_ resulted in activated carbons with significant surface areas and a high volume of micropores. AC-KOH exhibited a surface area of 1091 m^2^ g^−1^, with 83.33% of the micropore volume in the 0.7–1.0 nm range. In contrast, AC-KCO and AC-KHP displayed a larger volume of micropores in the 0.7–1.5 nm range (93.2% and 90.4%, respectively), with lower microporosity below 1.0 nm compared to AC-KOH. However, the micropore volume of AC-KHP in the 0.7–1.0 nm range (0.345 cm^3^ g^−1^) was higher than that of AC-KOH (0.333 cm^3^ g^−1^).

In TSA tests conducted at a temperature of 30 °C, a pressure of 1 bar, and a flow rate of 100 mL·min^−1^ (50% CO_2_ + 50% N_2_), the activated carbons AC-KOH, AC-KCO, and AC-KHP, produced from printed paper waste, adsorbed 7.64%, 6.28%, and 6.80% of CO_2_, respectively. These results demonstrate the effectiveness of activating agents, in contrast with the adsorption of only 1.98% observed in the reference carbon (without activation). The cyclic stability analysis of AC-KOH showed a minimal variation in CO_2_ adsorption capacity (average of less than 1%, between 7.60% and 7.55%) over ten adsorption–desorption cycles, indicating good cycle stability and regeneration performance. This research provides a valuable reference for the development of solid CO_2_ adsorbents using different types of paper found in municipal solid waste.

The activated carbons AC-KOH, AC-K_2_CO_3_, and AC-KHP exhibit a rapid initial adsorption rate due to the availability of active sites. However, as adsorption progresses, the occupation of these sites and the saturation of the pores by CO_2_ molecules increase the resistance to diffusion within the carbonaceous adsorbents. Equilibrium is reached in approximately 6 min for the three synthesized carbons, achieving adsorption capacities of 2.29 mmol g^−1^ (AC-KOH), 1.93 mmol g^−1^ (AC-K_2_CO_3_), and 2.03 mmol g^−1^ (AC-KHP).

## Data Availability

The authors declare that the data supporting the findings of this study is available within the paper. Alternative format versions of the raw data files are available from the corresponding author upon reasonable request.
